# A Review of Cognitive Hybrid Radio Frequency/Visible Light Communication Systems for Wireless Sensor Networks

**DOI:** 10.3390/s23187815

**Published:** 2023-09-12

**Authors:** Rodrigo Fuchs Miranda, Carlos Henrique Barriquello, Vitalio Alfonso Reguera, Gustavo Weber Denardin, Djeisson Hoffmann Thomas, Felipe Loose, Leonardo Saldanha Amaral

**Affiliations:** 1 Technology Center, Federal University of Santa Maria (UFSM), Santa Maria 97105-900, Brazil; rodrigo.miranda@acad.ufsm.br (R.F.M.); djeisson.thomas@ufsm.br (D.H.T.); leonardo.amaral@acad.ufsm.br (L.S.A.); 2 Automation and Intelligent Systems, ITR-Norte, Technological University of Uruguay (UTEC), Rivera 40000, Uruguay; vitalio.alfonso@utec.edu.uy; 3 Department of Electrical Engineering, Federal Technological University of Paraná (UTFPR), Campus Pato Branco, Pato Branco 85503-390, Brazil; gustavo@utfpr.edu.br; 4 Department of Electrical Engineering, Electronics, Communications and Systems, University of Oviedo (UNIOVI), 33204 Gijón, Spain; loosefelipe@uniovi.es

**Keywords:** cognitive radio, radio frequency, sensor networks, visible light communication, wireless communication

## Abstract

The development and growth of Wireless Sensor Networks (WSNs) is significantly propelled by advances in Radio Frequency (RF) and Visible Light Communication (VLC) technologies. This paper endeavors to present a comprehensive review of the state-of-the-art in cognitive hybrid RF-VLC systems for WSNs, emphasizing the critical task of seamlessly integrating Cognitive Radio Sensor Networks (CRSNs) and VLC technologies. The central challenge addressed is the intricate landscape of this integration, characterized by notable trade-offs between performance and complexity, which escalate with the addition of more devices and increased data rates. This scenario necessitates the development of advanced cognitive radio strategies, potentially facilitated by Machine Learning (ML) and Deep Learning (DL) approaches, albeit introducing new complexities such as the necessity for pre-training with extensive datasets. The review scrutinizes the fundamental aspects of CRSNs and VLC, spotlighting key areas like Energy Efficient Resource Allocation, Industrial Scenarios, and Energy Harvesting, and explores the synergistic amalgamation of these technologies as a promising pathway for enhanced spectrum utilization and network performance. By delving into the integration of cognitive radio technology with visible light, this study furnishes valuable insights into the potential for innovative applications in wireless communication, presenting a balanced overview of the current advancements and prospective avenues in the field of cognitive hybrid RF/VLC systems.

## 1. Introduction

Wireless Sensor Networks (WSNs) have garnered substantial attention due to their versatility in various sectors such as healthcare, industrial automation, and urban intelligence [1,2]. The surging demand for wireless communication services has precipitated a critical shortage of available Radio Frequency (RF) spectra. The traditional spectrum allocation policies have culminated in inefficient utilization, leaving certain frequency bands unused for extended periods. To counter this challenge, Cognitive Radio (CR) has emerged as a promising avenue, facilitating efficient and dynamic spectrum utilization [3,4].

Expanding upon the concept of CR, Cognitive Radio Sensor Networks (CRSNs) amalgamate CR technology with sensor networks, navigating the intricacies of dynamic and heterogeneous wireless environments. CRSNs combine the adaptability and intelligence of CRs with the sensing capabilities of sensor networks, optimizing spectrum usage, enhancing network performance, and fostering efficient data transmission. In CRSNs, sensor nodes endowed with CR capabilities autonomously sense, analyze, and adapt to the wireless environment, guided by real-time observations and application requirements [5,6].

CR augments wireless communication by adeptly identifying available communication channels and promptly transitioning to vacant ones, thus averting interference with Primary Users (PUs) who possess licensed spectrum rights [7]. This strategy, termed Dynamic Spectrum Access (DSA), permits Secondary Users (SUs) to opportunistically utilize underexploited frequency bands without inducing harmful interference, thereby optimizing the utilization of the RF spectrum. A complementary concept, Dynamic Spectrum Sharing (DSS), entails real-time spectrum sharing among multiple users, encompassing both PUs and SUs. Contrary to DSA, which centers on opportunistic access to underutilized spectrum bands, DSS dynamically allocates the spectrum to various users based on their immediate needs and the spectrum’s availability. This mechanism necessitates coordination and collaboration between PUs and SUs to safeguard efficient spectrum utilization while preserving the Quality of Service (QoS) for PUs [8,9].

Within a network of sensor nodes, CRSNs establish intelligent and adaptive wireless sensor networks. The cognitive functionalities of CR empower it to adapt and learn from the environment. Leveraging the integration of software-defined radio technology, CRs can be reprogrammed and reconfigured in real-time, facilitating the sensing, analysis, and response to alterations in the RF spectrum. This dynamic adjustment encompasses transmission parameters such as bandwidth, frequency, modulation, and power, optimizing communication performance while honoring the spectrum rights of PUs [10,11].

While numerous CR techniques have been devised to alleviate the congestion of overcrowded RF bands, innovative technologies have also been developed to address upper frequency bands, including the visible spectrum [12]. VLC has surfaced as a promising alternative, utilizing the visible light spectrum for wireless communication and employing Light-Emitting Diodes (LEDs) for data transmission. VLC presents several benefits, including high data rate transmission, broadband solutions, security features, unlicensed channels, and compatibility with 5G and 6G networks [13,14]. Moreover, VLC is immune to electromagnetic interference generated by RF devices, a trait that, coupled with other technical characteristics like the wide bandwidth available, positions VLC as a promising technology for critical communication environments [15,16].

The contribution of this paper is twofold. Firstly, we introduce an innovative framework for the seamless integration of CRSNs and VLC technologies, proposing a novel approach to harness the combined potential of these domains. This integration framework amplifies network efficiency and mitigates spectrum scarcity challenges. Secondly, the paper offers invaluable guidance for researchers and practitioners by pinpointing key open problems and research trajectories in the CRSNs and VLC integration domain. By tackling these challenges, we aspire to spur innovation and accelerate the evolution of next-generation wireless communication networks.

In the ensuing sections of this paper, we will delve deeply into the foundational principles of CRSNs and VLC, exploring their intricate interplay. We will also scrutinize the concept of heterogeneous networks and hybrid systems within environments equipped with advanced CR capabilities. Additionally, our discourse will encompass guidance for research and addressing open problems in the field, furnishing valuable insights for researchers aiming to advance the state of knowledge. To this end, we will present a contemporary review of the literature, underscoring the pivotal role VLC assumes in enhancing spectrum utilization and network performance within CRSNs contexts.

## 2. The Basics of CRSNs

CRSNs operate within intricate physical environments, where the adept management of various physical domains is critical to their performance. The vital physical domains in CRSNs encompass spectrum resources, time resources, power resources, and space resources.

- Spectrum resources: The range of electromagnetic frequencies employed for wireless communication is of paramount importance in CRSNs. As the spectrum hosts multiple critical applications, it is tightly regulated. CR is distinctive in its ability to dynamically access and utilize underutilized spectrum segments, known as “white spaces” or “spectrum holes”. The ability to intelligently detect unoccupied communication channels and adapt spectrum usage enables CR to coexist efficiently with other wireless systems.
- Time resources: Time synchronization is integral to CRSNs, as it facilitates coordination among sensor nodes and optimizes resource allocation. Through precise time alignment, CRSNs mitigate collisions and ensure efficient communication.
- Power resources: Power management is vital in CRSNs due to the limited energy resources of sensor nodes. By dynamically adjusting transmission power and routing paths, CR optimizes energy consumption, prolongs network lifespan, and enhances energy efficiency.
- Space resources: CRSNs leverage the spatial characteristics of wireless environments to optimize resource allocation and interference management. The strategic deployment of sensor nodes based on their physical locations aids in maximizing spectrum usage and enhancing network performance.

**Figure 1:**
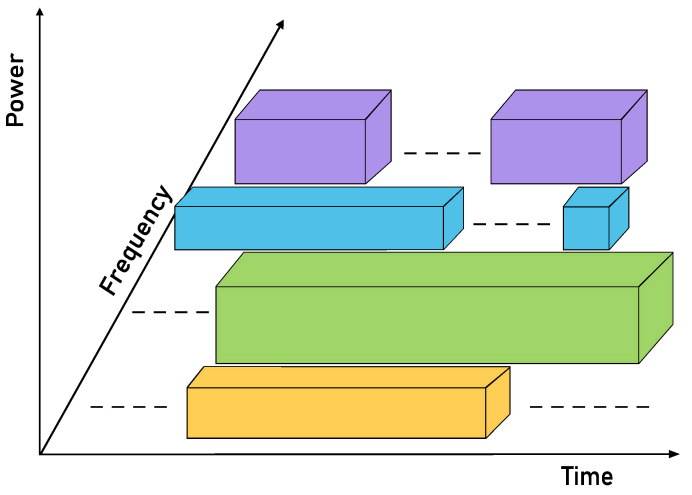
Example of the utilization of resources in a CRSNs.

An example of the utilization three of these resources in a CRSNs is illustrated in Figure 1.

In this figure, each color block represents the utilized resources of different PUs, and the hashed line represents the spectrum holes where the DSA can allocate the SUs.

Different strategies for managing these resources can be combined in CRSNs to optimize their operation in dynamic wireless environments. At the heart of CR lies spectrum management, a strategy comprising techniques that enable efficient spectrum allocation and utilization. Some key elements are listed below:

1. Spectrum sensing: CR devices detect unused or underutilized frequency bands;
2. Spectrum decision: based on sensed spectra, CR devices select the most suitable frequency bands for transmission;
3. Spectrum sharing: spectrum sharing strategies, including underlay, overlay, and hybrid, manage the allocation and sharing of the spectrum between PUs and SUs;
4. Interference mitigation: techniques such as power control mechanisms and adaptive modulation and coding schemes are employed to mitigate interference;
5. Spectrum mobility: CR devices utilize spectrum mobility techniques to efficiently use the available spectrum resources in dynamic spectrum environments;
6. Spectrum management database: a centralized repository of spectrum utilization data which informs about the availability of spectrum resources;
7. Dynamic spectrum Access: allows CR devices to dynamically adapt frequency usage based on real-time conditions and spectrum availability;
8. Spectrum monitoring and enforcement: involves continuous spectrum monitoring to maintain its integrity and ensure fair and efficient use.

**Figure 2:**
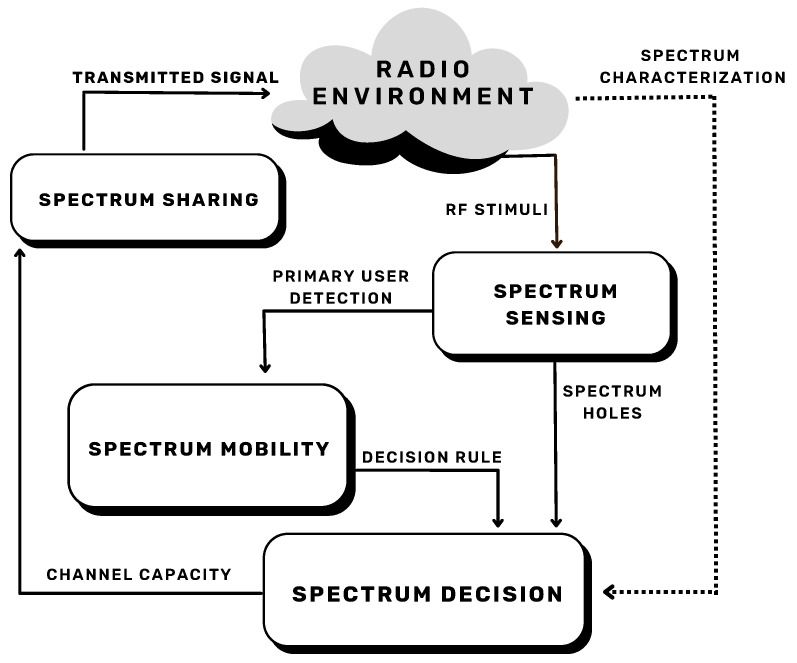
The CR cycle.

Figure 2 illustrates an example of the interaction of some main CR techniques. This is one of many possible examples of CR structure, also known as CR cycle, which helps to set the adaptive operation of cognitive capability [17,18,19].

These techniques aim at optimizing spectrum utilization, sharing resources among different users, maximizing spectrum efficiency, and ensuring fair and interference-free coexistence. They represent many aspects of managing the radio spectrum and enabling efficient access and utilization. Analogous strategies are applicable to the visible light spectrum, making them vital for hybrid communication systems that use both RF and VLC.

In the realm of hybrid RF/VLC systems, understanding and evaluating performance is paramount. The combination of RF and VLC technologies offers unique advantages, but also presents challenges that require careful analysis. Performance metrics provide essential insights into the system’s efficiency, reliability, and quality, guiding the design and optimization of these complex networks. In the following section, we will delve into key performance metrics that are particularly relevant to hybrid RF/VLC systems, highlighting their significance in the context of spectrum management and utilization.

### 2.1. Performance Metrics

CRSNs, as intricate systems combining the adaptive intelligence of CR with the multi-node potential of sensor networks, necessitate the assessment of diverse performance metrics.

Performance metrics are essential in CRSNs, as they provide a quantitative means to evaluate multiple aspects of the network. They guide decision-making for network optimization, resource allocation, proactive fault detection, and Quality of Service (QoS) monitoring. The selection of specific metrics for in-depth analysis is based on their relevance to hybrid RF/VLC systems [20,21,22].

#### 2.1.1. Fairness

This gauges the equitability of resource allocation among users or nodes. Fairness in resource allocation typically means that each user or device in the network should receive a fair share of the available resources. Fairness can be defined in various ways, depending on the specific goals and requirements of the network. Common fairness metrics include proportionally fair allocation, max-min fairness, and Jain’s fairness index, among others [23]. This metric is often evaluated using mathematical models [24].

Achieving fairness in resource allocation often involves a trade-off with network efficiency. Ensuring that every user receives a fair share may result in suboptimal resource utilization. Therefore, network operators and designers must strike a balance between fairness and efficiency based on the specific network goals and user requirements.

Fairness metrics become especially important in dynamic and heterogeneous wireless environments where users have varying channel conditions, QoS requirements, and traffic patterns. Adaptive resource allocation algorithms are often used to address these challenges [25,26].

#### 2.1.2. Outage

This serves as a metric for network reliability, indicating the chance of a wireless link’s quality falling below an acceptable level [27]. This implies the disruption or interruption in the normal functioning of a communication system or network, resulting in a temporary loss of connectivity or service. Outages can be caused by various factors, including hardware failures, software glitches, environmental conditions, interference, or deliberate attacks. Managing and mitigating outages are essential aspects of ensuring the reliability and performance of communication systems and networks.

Outage management in CRSNs is essential for maintaining reliable communication, data integrity, and network performance, particularly in dynamic and challenging wireless environments. Effective outage management strategies encompass adaptive routing, spectrum management, energy-efficient operation, and robust security measures to ensure that CRSNs continue to function even in the face of disruptions [28,29,30].

#### 2.1.3. Sum Rate

This represents the total maximum achievable data rates of all individual channels, providing a theoretical perspective of the network’s potential performance [31].

The sum rate metric in CRSNs quantifies the collective network capacity to transport information among sensor nodes while considering spectrum availability, interference management, and cognitive radio adaptation. Maximizing the sum rate in CRSNs is a pivotal objective, as it directly correlates with the network’s ability to efficiently utilize available resources, support various applications, and ensure reliable data exchange among sensor nodes in dynamic and spectrum-constrained environments [32,33].

#### 2.1.4. Throughput

This is the real rate of successful data delivery over the channel, presenting a more realistic performance measure compared to the theoretical sum rate. It accounts for the effective data transfer rate, accounting for factors like packet loss, retransmissions, and any protocol overhead [22,34].

In CRSNs, throughput is a critical performance metric as it directly reflects the network’s capacity to deliver data efficiently from source to destination. Maximizing throughput is essential for ensuring timely and reliable data communication among sensor nodes, particularly in applications where real-time data collection and exchange are vital. Achieving high throughput in CRSNs often involves optimizing resource allocation, spectrum utilization, and routing strategies to overcome challenges such as interference, fading, and dynamic spectrum access, all of which are common in cognitive radio environments [29,30,35].

#### 2.1.5. QoS

This aims to meet specific service requirements, which is particularly important in various applications, including voice and video communication, real-time monitoring, and critical data transmission. QoS ascertains the service level provided to users or nodes within a CRSNs, managing network resources and controlling performance characteristics [20,36].

QoS is typically measured using various metrics, including latency (delay), jitter (variation in latency), packet loss rate, and throughput (data transfer rate). These metrics help assess the overall quality and performance of a network or service.

In the context of CRSNs, QoS is vital to provide efficient and reliable data transmission, especially in scenarios with diverse traffic types, dynamic spectrum availability, and resource-constrained sensor nodes. By implementing QoS mechanisms, CRSNs can effectively adapt to changing conditions, optimize resource usage, and support a wide range of applications while meeting their specific QoS requirements [37,38].

## 3. The Basics of VLC

VLC is a communication methodology that utilizes the visible light spectrum within the frequency range of 400 to 800 nm. LEDs, extensively utilized in domestic, office, and public spaces, serve as the primary facilitators for VLC, offering both illumination and data transmission concurrently. A significant advantage of VLC is its non-reliance on the increasingly congested electromagnetic spectrum, thus positioning it as a viable system choice, particularly considering the rapid proliferation of wireless communication devices.

VLC operates by modulating the intensity of the LED light source to encode data. This modulation occurs at a high frequency, which is imperceptible to the human eye. At the receiving end, a photodiode detects this modulated light, converting the fluctuations in light intensity into an electrical current, which is subsequently transformed back into a binary data stream. Thus, the principle of operation is akin to data transmission through optical fibers, with the crucial distinction being that VLC employs free space for data transmission [39].

Traditional RF systems, although prevalent, are fraught with challenges. RF spectrum resources are not only scarce, but also licensed, resulting in spectrum congestion and interference issues. The deployment cost of RF-based systems can be substantial, and these systems often lack intrinsic security features necessary for certain applications. Moreover, RF systems necessitate substantial energy consumption, implying potential environmental ramifications.

In contrast, VLC offers several key advantages:

- Spectrum availability: VLC, categorized under free-space optical communication, provides an expansive spectrum, offering substantially larger bandwidth than the RF spectrum utilized by conventional wireless technologies [40];
- Resistance to RF interference and overcrowding: VLC is immune to electromagnetic interference that can disturb sensitive electronic systems. This unique property enables VLC to function even in environments where RF usage is restricted, such as hospitals, airplanes, power plants, and sensitive industrial settings [41];
- Cost-effective implementation: VLC leverages readily available, cost-effective LEDs. Existing infrastructures can be conveniently adapted for VLC by incorporating LEDs into the existing lighting systems, thereby providing concurrent communication and illumination. This is particularly beneficial in scenarios such as vehicular communication where LED lights are already in operation, and introducing RF systems would entail additional equipment and associated costs [42,43];
- Enhanced security: The physical properties of visible light, such as its inability to penetrate solid objects and walls, inherently enhance wireless communication security. Moreover, the visibility of the light facilitates easy identification of potential receivers [44,45];
- Energy saving and potential environmental impact: VLC utilizes existing LED lighting systems, which can be more energy-efficient compared to some radio wave technologies, depending on the specific implementation and energy source. LEDs might also offer certain environmental advantages, such as potentially reduced use of hazardous materials in their design and possibly lower heat generation, although these benefits may vary widely based on the particular technology, application, and lifecycle considerations [46,47].

Despite these advantages, VLC has its own set of challenges:

- Line-of-Sight (LoS): VLC generally necessitates a clear LoS between the transmitter and receiver, potentially limiting its applicability in certain situations. This requirement also introduces constraints on mobility and coverage [48,49].
- Distance range: The range of VLC is largely dictated by the power of the light source signal and the sensitivity of the receiver. The presence of obstacles and diverse environmental factors can further curtail range by blocking or degrading the light signals [50].
- Interference: Interference, a significant challenge in VLC, can disrupt data signal transmission. Interference can originate from ambient light sources (both natural and artificial), cross-talk among VLC systems, multi-path interference due to reflections and scattering, and other light sources, all of which can impact the reliability and quality of VLC communication [51].
- Uplink challenges: The uplink, crucial for transmitting collected data from sensor nodes to a central controller or sink node, poses challenges in VLC systems due to weaker signal strength, irradiance interference from ambient light sources, limited receiver sensitivity and bandwidth, LoS requirements, and mobility constraints. These factors hinder the achievement of efficient and reliable uplink transmission [52].
- Outdoor environment: VLC is vulnerable to atmospheric turbulence, leading to signal fading and degradation. It confronts multiple challenges such as elevated noise levels, interference, and restricted mobility owing to the requirement for a LoS configuration. Moreover, the SNR is adversely affected by environmental conditions like fog, rain, snow, dust, haze, and sunlight [53]. The technology also grapples with limited transmission range and the intricacies of integration with other communication systems, such as RF. Furthermore, there is a necessity for specialized modulation and coding schemes. These constraints render outdoor VLC less versatile than its indoor counterpart [54,55].

While VLC has applications in both indoor and outdoor environments, the primary focus of this review is on its indoor applications. The rationale behind this focus is twofold. First, the subject of VLC is already broad and encompasses a wide range of research areas, making it necessary to narrow down the scope for a more in-depth analysis. Second, we aim to discuss Free Space Optics (FSO), another optical wireless communication technology, albeit briefly, which is more suited for outdoor environments. Therefore, the review will largely concentrate on the indoor aspects of VLC and integration with RF technologies and cognitive strategies.

In the context of Internet of Things (IoT) applications, VLC plays a pivotal role, finding applications in indoor positioning systems, vehicle-to-vehicle communication, and underwater communication. Given these reasons, many IoT devices operate on batteries and, hence, the energy efficiency of VLC represents an additional advantage. Moreover, VLC’s immunity to RF interference is critical in IoT environments, where such interference can disrupt sensitive electronic systems.

Furthermore, CR can manage the spectra of both RF and VLC concurrently, offering a more efficient spectrum management approach. Therefore, as a shared resource similar to RF, VLC can also be managed by CR in order to optimize spectral utilization, reduce interference, and enhance overall efficiency in complex communication environments.

## 4. Heterogeneous Network (HetNet)

Heterogeneous Networks (HetNets) are integral infrastructures constituting a strategic intersection of numerous communication technologies and diverse base stations. Their main objectives include expanding coverage, enhancing capacity, and improving the overall system performance. Essentially, HetNets merge multiple cellular typologies, crafting a seamless, high-efficiency network infrastructure.

On the other hand, within the HetNets, hybrid systems denote the harmonious fusion of two or more distinctive network types. These systems’ configuration relies on the unique network typologies and respective application use-cases. A hybrid system can function as an uplink, a downlink, or both, depending on the specific requirements. Essential considerations for hybrid systems include physical layer security, network selection, load distribution, mobility, scheduling, protocols for uplink/downlink, and resource sharing. An integrated resource allocation mechanism can be established to distribute bandwidth and power between the constituent networks.

Within the framework of hybrid networks, the uplink and downlink dichotomy depends on the employed wireless technology and the strategic application blueprint. Given the lower power tolerance of LED-based receiver devices, which is due to their sensitivity to high power inputs potentially leading to saturation, RF systems emerge as the preferred choice for uplink communication in hybrid RF/optical systems. Both RF and optical systems provide viable options for downlink communication. Optical Wireless Communication systems are often utilized for high data rate transmissions with LOS, whereas RF downlink finds its application in overcoming interference, Non-Line-of-Sight (NLOS) limitations, and facilitating handovers.

**Figure 3:**
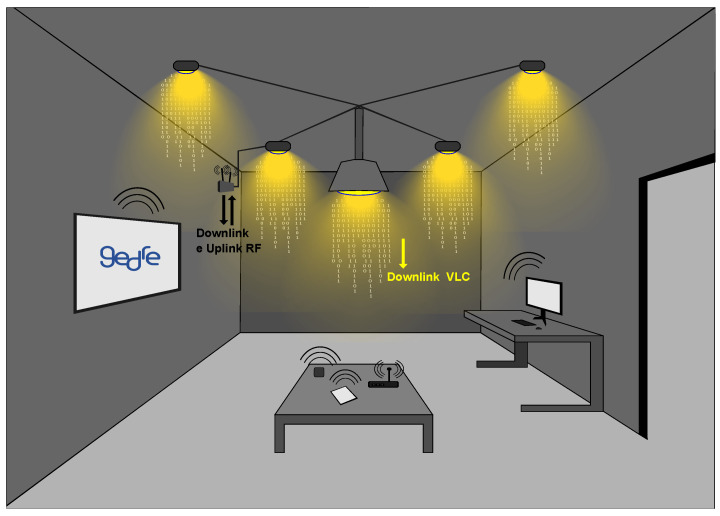
Illustration of a hybrid RF/VLC network in an indoor scenario.

To illustrate a HetNet, consider a hybrid RF/VLC system within an indoor scenario. In Figure 3, RF is employed for both uplink and downlink communication, while VLC is used exclusively for downlink communication.

This network can be configured in many other ways, depending on the application requirements [31]. These configurations include:

- Dual-hop hybrid RF/VLC system: this system operates in two hops; the first hop is RF for long-distance communication, and the second hop is VLC for short-distance communication;
- Opportunistic separate networks (RF/VLC): these networks operate RF and VLC independently, selecting the network opportunistically based on network conditions and user requirements;
- Heterogeneous Networks (HetNets) with a centralized unit: These networks comprise RF and VLC networks with a centralized control unit, which assigns resources to the users based on the network conditions. This network is needed when a high number of users are present in a small area network.

The incorporation of CR networks, characterized by their ability to dynamically adapt to the spectrum environment, into hybrid RF/VLC systems can provide several benefits. CR networks, characterized by intelligent radios, are capable of dynamically utilizing the available spectrum resources. By integrating RF and VLC technologies within CRs, these networks can adaptively select the optimal channel according to prevailing conditions, thereby promoting efficient spectrum utilization, improved quality of service, and enhanced network capacity. Moreover, the complementary attributes of RF and VLC offer numerous benefits within CR networks, such as:

- Spectrum utilization: the unique frequency ranges of RF and VLC allow hybrid RF/VLC systems to promote efficient spectrum utilization;
- Coverage and mobility: RF and VLC provide complementary characteristics in coverage and mobility;
- Interference mitigation: by integrating RF and VLC, hybrid systems can offload communication tasks to VLC in high RF interference areas, thereby ensuring reliable communication;
- Data offloading and resource management: hybrid RF/VLC systems facilitate efficient data offloading and resource management;
- Reliability and adaptability: the complementarity of RF and VLC in hybrid systems enhances their reliability and adaptability.

Moreover, there are specific system requirements for implementing a hybrid RF/VLC wireless communication system, particularly in an indoor environment. These requirements include:

- Noise and multipath propagation: hybrid systems must address noise and multipath propagation phenomena in specific home environments to ensure reliable communication;
- Non-determinism in wireless communication: hybrid systems should strive to eliminate as many sources of non-determinism as possible;
- Co-existence with multiple wireless units: hybrid systems should coexist with other wireless units without causing or suffering interference;
- Backward and forward compatibility: hybrid systems should ensure compatibility with both older and newer devices and technologies;
- Fault tolerance: hybrid systems should be able to tolerate faults and prevent performance degradation.

By addressing these system requirements, a hybrid RF/VLC wireless communication system can be effectively implemented in an indoor environment. This ensures reliable and efficient communication despite the challenges posed by noise, multipath propagation, non-determinism, coexistence with other wireless units, compatibility with multiple devices, and resilience to faults.

One application example that can take advantage from HetNets is indoor positioning systems. The integration of RF and VLC technologies offers a multifaceted approach to localization. One study [56] employs Cramer–Rao Lower Bound (CRLB) metrics, a statistical measure that quantifies the lower bound of the variance of unbiased estimators, to demonstrate that hybrid systems can achieve superior positioning accuracy, formulated as:

(1) $${CRLB}_{Hybrid}<\min\left({CRLB}_{RF},{CRLB}_{VLC}\right).$$

This is complemented by the device-free localization advantages highlighted in another study [57], which suggests that hybrid systems can enhance user privacy while reducing hardware requirements.

A study on Channel State Information (CSI)-based systems [58] elucidates methods to mitigate the challenges posed by multipath fading. Specifically, it mentions that the multipath effect is suppressed and the positioning accuracy is improved by utilizing CSI. The paper also highlights the fusion of angle and range approaches, achieving a median error of 0.6 m in static environments and less than 2 m accuracy with 80% confidence in an office setting.

On the VLC side, challenges related to LoS are discussed, which can be mitigated in hybrid systems [59]. Another study [60] specifically discusses the trade-offs between precision and implementation complexity in VLC-based systems. It notes that the system is sensitive to ambient light noise and the alignment of the ultrasonic sensors, but benefits from a simple structure built with low-cost commercial components.

Incorporating cognitive strategies, a study [61] introduced the concept of light flicker fingerprinting, a technique that utilizes the unique light flicker patterns of LED bulbs for localization. The paper reports that the individual house can be identified with an accuracy of up to 86.3%, 93.6%, and 67.9% for normal, noisy, and full datasets, respectively. Furthermore, the paper employs k-Nearest Neighbors (kNN), Principal Component Analysis with k-Nearest Neighbors (PCA-kNN), and Principal Component Analysis with Neural Networks (PCA-NN) as classification methods, achieving an individual bulb identification accuracy across all houses of 24.9%, 23.9%, and 41.6%, respectively. These metrics not only demonstrate the system’s adaptability but also its performance in complex indoor environments.

## 5. Hybrid Systems Review

In conducting the literature review for this study, a systematic search was undertaken across several renowned databases, including Google Scholar, IEEE Xplore Digital Library, Multidisciplinary Digital Publishing Institute (MDPI), The International Society of Optics and Photonics (SPIE), Wiley Online Library, arXiv, Optica Publishing Group, Scopus, and Springer. The search strategy employed the use of specific keywords, namely “cognitive radio”, “hybrid RF/VLC”, and “cooperative schemes” (as a potential variation associated with cognitive radio), which were applied to the title, abstract, and keywords sections of potential articles. The temporal scope of the search was confined to the period between 2013 and 2023.

Many survey papers have discussed Hybrid Systems, particularly focusing on the integration of RF and VLC technologies. However, these papers often lack a comprehensive discussion on the integration of cognitive radio technology and wireless sensor networks, which is the primary focus of our review. A comparative summary of these survey papers is provided in Table 1.

CR strategies, originally formulated for RF networks, can be tailored and employed for VLC systems, subject to the unique attributes inherent to VLC. This section delineates the key disparities between RF and VLC and outlines the potential modulations needed for VLC compatibility.

- Spectrum use and management: In contrast to the regulated RF spectrum, VLC operates on the visible light segment of the electromagnetic spectrum, which is unlicensed and devoid of regulatory constraints. The visible light spectrum surpasses the RF spectrum in terms of bandwidth, thereby offering a stronger data transfer capacity. VLC systems typically utilize LED light sources, modulating the light intensity to encode and convey data.
- Propagation characteristics: Light waves, unlike RF waves, cannot permeate walls or other opaque structures, necessitating VLC to predominantly rely on LoS communication. This mandates that the transmitter and receiver maintain a direct visual pathway. Nonetheless, the propensity of light to reflect off surfaces enables NLoS communication. For instance, a CR-inspired approach could dynamically optimize the VLC system parameters in response to prevailing propagation conditions. Additionally, CR tactics for mitigating multi-path fading, such as employing diversity techniques, can be adapted to handle reflections in VLC systems [67].
- Interference management: VLC systems are susceptible to interference from many light sources, including sunlight, incandescent lamps, and other LED units. This interference can be curtailed by implementing filters or similar techniques to attenuate its effects [68]. Cooperative communication in VLC might encompass sharing interference source data and coordinating transmissions to circumvent interference. Furthermore, adaptive power control can be harnessed to adjust the VLC light source intensity according to the ambient light conditions.
- Security concerns: The security measures in VLC systems are distinctive due to their unique transmission medium. The communication is restricted within a confined space, limiting the possibility of eavesdropping, and hence enhancing the security.
- Spatial reuse: The inability of light to infiltrate through walls empowers the same frequency (or color) to be deployed for communication in adjacent spaces without inciting interference. This principle, known as spatial reuse, can be further enhanced in a cooperative VLC environment through judicious frequency coordination.
- Beamforming and MIMO: Beamforming, a technique refined in RF communication to concentrate signals towards specific antennas, can be implemented in VLC through the focus or shape modulation of the light beam. Likewise, Multiple-Input Multiple-Output (MIMO) techniques, employing multiple transmitters and receivers for performance enhancement, are transferable to VLC systems. Nonetheless, the actualization of beamforming and MIMO in VLC might require modification due to the distinct propagation characteristics of light.
  Cooperative communication in VLC could involve the strategic positioning and orientation of devices to maximize the benefits of both LoS and NLoS communication paradigms.

In the efforts to group the key areas where CR strategies can improve hybrid RF/VLC network, we have identified the following topics in Table 2.

### 5.1. Hybrid RF/VLC

The two review papers, referenced as [31,62], offer extensive insights into hybrid RF/VLC systems, albeit with distinct focal points and applications.

The first paper, [31], primarily concentrates on the architectures and key technologies underpinning hybrid RF/VLC systems. It elucidates the integration of RF and VLC technologies, the merits of hybrid systems, and the challenges encountered in their deployment. The paper underscores the potential of cognitive radio technology in enhancing the performance of hybrid RF/VLC systems and further delves into the application of machine learning techniques for intelligent decision-making within these systems. The key topics encapsulated in this paper include:

- Hybrid RF/VLC system architectures: the paper discusses three types of hybrid RF/VLC network topologies, namely the dual-hop hybrid RF/VLC system, opportunistic separate networks (RF/VLC), and heterogeneous networks (HetNets) with a centralized unit;
- Cognitive strategies: the paper provides an in-depth analysis of cognitive strategies applied in hybrid RF/VLC systems, including spectrum sensing, spectrum management, and spectrum mobility;
- Machine learning applications: the paper discusses the application of machine learning techniques in hybrid RF/VLC systems, including reinforcement learning, deep learning, and federated learning.

Conversely, the second paper, [62], provides a more comprehensive perspective on hybrid wireless networks. It discusses the practical considerations, challenges, applications, and future research directions of these networks. The paper emphasizes the importance of hybrid RF/VLC systems in achieving high-speed, reliable, and secure wireless communication. It further discusses the potential of these systems in various applications, including vehicular communication, indoor positioning, and IoT. The key topics covered in this paper include:

- Practical considerations: the paper discusses the practical considerations in implementing hybrid wireless networks, including noise and multipath propagation, non-determinism in wireless communication, co-existence with multiple wireless units, backward and forward compatibility, and fault tolerance;
- Challenges: the paper provides an in-depth analysis of the challenges faced in implementing hybrid wireless networks, including interference mitigation, data offloading and resource management, and reliability and adaptability;
- Applications: the paper discusses the potential applications of hybrid wireless networks, including vehicular communication, indoor positioning, and IoT.

In summary, both papers offer comprehensive reviews on hybrid RF/VLC systems, albeit with different emphases. The first paper accentuates the technical aspects and potential of cognitive radio and machine learning technologies in hybrid systems. In contrast, the second paper provides a broader perspective on the practical considerations, challenges, and applications of hybrid wireless networks.

### 5.2. Free Space Optics (FSO)

In the field of FSO and hybrid VLC/RF systems, several studies offer unique contributions. Paper [91] introduces new mathematical models for outage probability, showing that the diversity of order is influenced by the fading severity of the RF link and turbulence parameters of the FSO link, but is independent of primary user parameters. Papers [92,93] achieve a remarkable 92% accuracy in real-time user identification in FSO systems through machine learning. Paper [94] introduces a spectrally efficient cognitive radio RF/FSO scheme, showing that system performance is influenced by weather conditions and the number of relays. Paper [95] presents a novel multi-QoS system design that optimizes the number of medium-priority links supported by a single RF resource. Papers [96,97] provide new mathematical models for performance metrics and demonstrate that adjusting the interference threshold can improve bit error probability performance significantly. Finally, paper [98] employs decode-and-forward based cooperation with multiple antennas, showing improved outage performance compared to simpler systems. Collectively, these papers advance the field by offering specific contributions in areas such as diversity order, user identification accuracy, environmental factors affecting system performance, resource allocation, and cooperative strategies.

### 5.3. Energy Efficient Resource Allocation

Energy efficiency is a paramount consideration in CRSNs, as sensor nodes typically operate on battery power and possess limited energy resources. Various key aspects play instrumental roles in achieving energy efficiency, including power control techniques, sleep scheduling strategies, resource allocation strategies, energy-aware routing protocols, DSA, energy harvesting techniques, and cross-layer optimization techniques.

This subsection examines six papers that propose different systems and optimization methodologies for energy-efficient resource allocation in hybrid Radio RF/VLC networks.

In [76], the focus is on designing a system that can provide energy-efficient resource allocation in a hybrid RF/VLC network. The proposed system utilizes an online algorithm responsive to current network conditions. This responsiveness facilitates dynamic adjustments to resource allocation, reducing overall power consumption. A crucial component of this system is its capability to ensure all users remain connected to at least one Access Point (AP) at all times, enhancing network reliability. However, the complexity of the Integer Linear Programming (ILP) algorithm used in this system presents a challenge, especially in the context of large-scale networks. The algorithm’s high demand for computational resources may limit its real-time applicability in scenarios requiring rapid adjustments to resource allocation.

The cognitive strategy implemented in this study is not explicitly mentioned. However, the use of an online algorithm that dynamically adjusts the allocation of resources based on the current network conditions, thereby reducing the overall power consumption. Secondly, the system ensures that all users are connected to at least one AP, which enhances the reliability and robustness of the network. The APs are controlled by a central controller that decides which APs should be turned on based on the illumination requirements and user requests, and this strategy suggests a type of cognitive functionality.

Paper [75] delves into the complexities of resource allocation in hybrid VLC and RF networks that share a common backhaul. An optimization framework that ensures fair allocation of resources, such as power, bandwidth, and time, between the RF and VLC subsystems is proposed. This framework is designed to maximize the corresponding data rate while maintaining fairness among the users, a crucial aspect in such heterogeneous networks where a standard rate maximization approach could lead to a severely degraded performance for the weaker users. The authors transform the non-convex problem into an equivalent convex one to provide a tractable solution. They also present a simplified power allocation problem that offers comparable results with significantly reduced complexity. The paper further discusses the impact of imperfect CSI on the overall network performance, and proposes a simplified resource allocation framework based on equal distribution of time and bandwidth resources to users. This comprehensive analysis of resource allocation is particularly relevant when dealing with the challenges posed by networks sharing a common backhaul.

Again, the cognitive element is not explicitly stated in this paper. However, the transformation of the non-convex problem into an equivalent convex one indicates a form of cognitive problem-solving. The algorithm assesses the nature of the problem and adapts its approach to make it more tractable. Furthermore, the consideration of imperfect CSI implies that the system must intelligently adapt to variations in channel quality.

In [70] the authors explore the application of cognitive technology in scenarios involving multiple LEDs, aiming to maximize the sum rate. They supplement the data rate not achieved by the VLC system with the RF system, enhancing energy efficiency, total data rate, and sum rate.

The proposed cognitive strategy is based on a mathematical model that optimizes resource allocation within the network. The model considers factors such as the number of LEDs, LoS availability probability, and total communication power.

The problem is formulated as a convex optimization task, a concave-convex fractional program, with an objective to optimize the energy efficiency of the heterogeneous network. This is achieved by allocating appropriate transmission powers and bandwidths to mobile terminals. The Karush–Kuhn–Tucker (KKT) conditions are used for determining the optimal power allocation.

The paper demonstrates two operational modes - underlay and overlay. The power allocation to the primary user (PU) is based on channel gain in both modes, while the power to the secondary user is influenced by both the channel gain and the power given to the PU.

Despite the complexity of the problem and the reliance on simulations, the system offers improvements in energy-efficient resource allocation for mixed RF/VLC networks. The authors suggest future work on the coordination between access points, association of mobile terminals to an access point, and the effects of interference.

In conclusion, the integration of VLC and RF APs in heterogeneous wireless networks has shown to significantly improve energy efficiency, outperforming RF-only networks and heterogeneous networks comprising two RF systems.

The authors of [72] put forth a novel resource management approach within Industrial IoT networks. They present a heterogeneous RF/VLC industrial network architecture designed to accommodate diverse QoS requirements.

Industrial IoT, or Industry 4.0, encompasses the integration of IoT technologies within manufacturing and industrial practices. Industrial IoT devices, including sensors, machines, actuators, and robots, facilitate real-time industrial control with minimal human intervention by intelligently transmitting data. These devices are leveraged in a range of critical applications, from real-time monitoring and controlling of industrial processes, and predictive maintenance of machines, to the optimization of resource usage within an industrial setting.

The proposed RF/VLC network aims to cater to two groups of devices with distinct QoS needs: Group 1, necessitating Ultra-Reliable Low-Latency Communications (URLLC), and Group 2, requiring high data rates. For Group 1 devices, predominantly comprising Industrial IoT devices, ultra-reliable low-latency communication is indispensable, given their involvement in real-time industrial control applications. For instance, immediate reporting of critical changes in machine status by a sensor or instant transmission of instructions to an actuator by a central controller is essential. Significant delay or data loss in these contexts could instigate operational inefficiencies, equipment damage, or even safety risks.

The authors formulate the energy-efficient resource management challenge as a Markov Decision Process (MDP), encapsulating network selection, subchannel assignment, and power management. The objective is to enhance network energy efficiency while ensuring the QoS requirements of IoT devices are fulfilled. Emphasis is placed on satisfying the ultra-reliable low-latency demands of Industrial IoT devices.

Despite these advancements, the authors identify potential avenues for future research, such as the necessity for improved coordination between APs, especially in VLC networks. The paper further emphasizes the need to explore the association of Mobile Terminals to an AP, and the impact of interference. The authors suggest that future work could contemplate the development of optimal resource allocation algorithms for diverse modulation schemes.

The research paper [71] puts forth a cognitive model for VLC aimed at augmenting spectral efficiency. The model delineates users into primary and secondary categories, contingent upon their service requirements. Spectrum sensing is employed to ascertain user needs, safeguard designated users, and identify usable spectrum for optimal utilization. The results of this sensing process guide the selection of the appropriate scheduling scheme.

The study proposes a versatile hybrid underlay/overlay scheduling approach to cater to diverse users and bolster spectral performance. The sum-rate and spectral efficiency of users at the cell edge are assessed under diverse scheduling schemes. The cognitive strategy proves beneficial in enhancing the achievable sum-rate for cell edge users, contingent on the LED switching status.

The research concludes that the hybrid system surpasses the overlay and underlay models in terms of sum-rate and spectral efficiency. The FoV emerges as a crucial parameter that yields optimal results, as it enables users to fine-tune reception based on their location. The impact of reflection and transmission from the ceiling or walls within a room is also noted. NLOS conditions and mobile users are identified as significant challenges that could severely impair device performance.

Nonetheless, the paper does not offer a comprehensive solution to the NLOS issue and the influence of mobile users on device performance, indicating potential avenues for future research and enhancement in the field of cognitive VLC.

Paper [65] uses a hybrid RF/VLC network to improve connectivity in IoT, particularly in areas lacking high-speed radio coverage. The architecture is cellular, with each cell’s center being a lighting system powered by solar panels, which also powers the rest of the systems, contributing to the system’s sustainability.

The main challenge to this system is achieving a high level of connectivity between devices and the interoperability. To validate the proposed system, the authors conducted a series of experiments, including a fragmentation procedure test, VLC communication tests, and real-scenario experiments. These experiments involved sending data packets between endpoints at different distances and measuring transfer times. The tests demonstrated the system’s effectiveness and reliability in many scenarios and conditions.

In conclusion, the proposed hybrid RF/VLC network architecture presents a promising solution for enhancing connectivity in the IoT domain. Despite the challenges, its potential benefits, such as high connectivity, interoperability, and energy efficiency, make it a compelling area for further research and development. The conducted experiments provide a comprehensive validation of the proposed system.

Paper [79] unveils a groundbreaking approach to energy-efficient resource allocation in 5G networks, capitalizing on the integration of passive reflectors to bolster connectivity, particularly in areas marred by physical obstructions. This initiative is a significant leap in the ongoing efforts to refine resource allocation strategies in cognitive hybrid RF/VLC systems, promising advancements in network capacity and user mobility.

The authors tackle the challenge by formulating an optimization problem with the dual objective of maximizing the SNR and minimizing total power consumption. This process is orchestrated through a two-step algorithm that, firstly, identifies optimal locations for the passive reflectors and, secondly, optimizes power allocation at the base stations to enhance SNR. The Particle Swarm Optimization (PSO), a technique grounded in artificial intelligence, plays a pivotal role in this optimization, demonstrating a faster convergence speed compared to other techniques like the Genetic algorithm, thereby optimizing computational costs and enhancing energy efficiency.

Simulations reveal that this approach significantly elevates connectivity and energy efficiency compared to systems devoid of passive reflectors, indicating its potential to revolutionize resource allocation and network performance in environments with physical obstructions.

In conclusion, the techniques used in these six papers collectively demonstrate the expansive nature of research in energy-efficient resource allocation. However, the challenges identified underscore that further work is necessary in this area. Future research should focus on devising innovative strategies for energy conservation in CRSNs that consider the practical constraints and requirements of real-world applications. Moreover, efforts should be made to bridge the gap between theoretical optimizations and real-world implementation of these systems. As the field evolves, it will be crucial to continue examining diverse approaches and techniques to identify the most efficient and effective solutions. Table 3 provides a comparative summary of the six papers, detailing the proposed system, performance analysis, key metrics, and the type of problem formulation.

### 5.4. Industrial Scenarios

Industrial environments, characterized by a high prevalence of metallic machinery, often generate substantial electromagnetic interference. VLC, due to its robust resistance to such disturbances, outperforms and complements RF communications, known for their susceptibility to interference [80].

Incorporating cognitive strategies within this challenging landscape of interference and spectrum congestion can significantly enhance network performance. This lends credence to the implementation of hybrid RF/VLC systems in industrial settings, especially with the rising need for high-rate IoT communications. Consequently, the hybrid RF/VLC network emerges as a promising solution for designing flexible and high-performance communication systems tailored to industrial scenarios.

Paper [67] concentrates on VLC channel characteristics and its application in an industrial environment, proposing adaptive techniques for performance optimization. Similar principles of adaptability and optimization apply to both VLC and CRSNs. The cognitive strategies in this work encompass data collection, analysis, and interpretation for understanding VLC behavior in industrial settings. The paper considers several factors in utilizing VLC in industrial environments, including:

- Channel characteristics: Investigation of the VLC channel in IIoT scenarios was executed using a ray-tracing simulation method. The study analyzed and modeled large-scale fading and multipath-related characteristics through distance-dependent and statistical distribution models;
- Density of surrounding objects: The impact of surrounding object density on VLC channel characteristics was evaluated under a single transmitter. The findings suggest that users in denser environments receive more multipath components, resulting in reduced optical path loss and increased RMS DS;
- User heights: A comparison of large-scale fading and multipath-related characteristics was performed at different user heights under multiple transmitters. Results indicate increased optical path loss and reduced RMS DS at lower receiver heights;
- Link adaptation method: The study proposes a two-step link adaptation method combining luminary adaptive selection and delay adaptation to mitigate multipath interference. Simulation-based verification of this method showed optimization in several parameters such as SNR, RMS DS, Channel Impulse Responses, and BER of a DCO-OFDM system.

The two-step adaptation method comprises:

1. Luminary adaptive selection: a strategy utilizing a greedy algorithm to select the optimal LED index for maximizing SNR;
2. Delay adaptation technique: a strategy that controls effective signal transmission time, ensuring LoS components from variable transmitters arrive at the receiver simultaneously.

Despite the absence of explicit discussion on hybrid configurations, the adaptive techniques proposed can be paralleled with dynamic spectrum management strategies used in CR. Both systems adapt to their environments to enhance performance.

Paper [82] explores strategies for VLC performance enhancement, highlighting benefits in improving system throughput and providing fair coverage. Although the study does not explicitly focus on industrial environments, its proposals, including cooperative multipoint techniques and MIMO precoding, provide solutions to common challenges in these environments such as LoS blockage and interference.

The application of Massive MIMO (m-MIMO) techniques, discussed in this work, holds particular relevance for industrial environments. By serving multiple users simultaneously in the same frequency band, m-MIMO significantly improves spectral efficiency and throughput, making it an asset for industrial settings with multiple IoT devices.

M-MIMO also boosts communication system robustness, enhancing resistance to interference and blockages common in industrial settings. The dynamic resource management techniques discussed can adapt the VLC system to operational requirements, enhancing system efficiency and performance. However, the authors also highlight challenges such as limited backhaul links, predicting CSI for mobile users, achieving channel response among users, user-centric clustering schemes, and the applicability of m-MIMO to VLC.

### 5.5. Energy Harvesting

CRSNs traditionally rely on batteries or external power sources for operation. An alternative approach is energy harvesting, which captures energy from the environment, such as light, heat, vibrations, or radio waves, and converts it into electrical energy to power the CR devices.

VLC, a technology that transmits data through light, can integrate with indoor photovoltaics. Herein, the photovoltaic cells harness the light used for VLC, constructing a self-sustaining system. This conjunction of CR and VLC with indoor photovoltaics presents an exciting potential for developing fully autonomous, self-powered sensor devices, applicable across various sectors including industries, healthcare, home environments, and smart cities.

In [99], the authors propose a hybrid VLC-RF system for indoor IoT applications that uses a time-splitting-based light energy harvesting model. This model aims to capitalize on the light signal during the idle state of the downlink, with the harvested energy then utilized for RF uplink transmissions.

The paper outlines a carrier allocation VLC system wherein multiple RF carriers are utilized for signal modulation. Through photoelectric conversion, the receiver discriminates signals transmitted from different LED lamps, using bandpass filters. When a specific light (the *i*th light) is selected for information delivery to a specific device (the *j*th device), signals from the other lamps can be used for energy harvesting.

It is assumed that only one LED light is selected for data transmission over the downlink. In the time-splitting scheme, the light from the selected lamp will only be used for information decoding during the data transmission stage. Simultaneously, the light signal from the same lamp will be adopted for energy harvesting during the light energy harvesting stage.

The energy harvested during the light energy harvesting stage over the VLC downlink can be described as:

(2) $$E_{j,LEH}=0.75T_{ij,dn}\cdot Vt\cdot I_{DC}^{2}\cdot L^{2}\cdot \rho_{j}\left(1-\eta_{ij}\right)/I_{0}.$$

where:

- $T_{ij,dn}$ denotes the downlink transmission time from LED *i* to device *j*;
- Vt is the thermal voltage of the photodetector;
- $I_{DC}$ signifies the DC component of the output current from the photodetector;
- *L* represents the side length of a room model;
- $\rho_{j}$ indicates the energy harvesting efficiency at the *j*th device;
- $1-\eta_{ij}$ stands for the proportion of the room’s side length not covered by the distance from the LED to the device;
- $I_{0}$ is the dark saturation current of the photodetector.

This model allows energy to be harvested from the light signals transmitted by LED lamps, which can then power the devices in the system. The time-splitting-based model uses the idle state of the downlink, when there is no active data transmission, to fully utilize the available light energy without interfering with the communication process.

A significant advancement towards sustainable energy management for IoT devices is presented in [100], where high-efficiency ambient photovoltaics, powered by ambient light, achieved a power conversion efficiency of 38% at 1.0 V open-circuit voltage at 1000 lux (fluorescent lamp). This development is particularly impactful for indoor environments with a prevalence of IoT devices and signals the potential for indoor photovoltaics to generate fully autonomous, self-powered sensor devices.

### 5.6. Simultaneous Wireless Information and Power Transfer (SWIPT) and Simultaneous Lightwave Information and Power Transfer (SLIPT) Incorporation in CRSNs

CR networks have emerged as a promising solution to the pressing dual challenge of energy efficiency and optimal spectrum utilization. This is particularly apparent with the integration of SWIPT and SLIPT techniques. These techniques enable the concurrent transfer of data and power over a single wireless channel, thereby enhancing the operational efficiency of CR networks.

SWIPT, primarily deployed in RF systems, involves the transmission of a signal imbued with both information and power. At the receiver, an energy harvester is employed to extract power from the received signal—a function of both the signal’s strength and the harvester’s efficiency. Concurrently, a separate decoder is used to decode the transmitted information. The power transfer in RF-based systems, such as SWIPT, can be computed using the Friis transmission equation:

(3) $$P_{r}=P_{t}+G_{t}+G_{r}+20\log\left(\frac{\lambda }{4\pi d}\right).$$

where $P_{r}$ denotes the power received, $P_{t}$ the power transmitted, $G_{t}$ the gain of the transmitting antenna, $G_{r}$ the gain of the receiving antenna, λ the wavelength of the carrier signal, and *d* the distance between the transmitter and receiver.

In contrast, SLIPT is analogous in principle but finds its application in VLC systems. Here, light (commonly sourced from Light Emitting Diodes (LEDs)) substitutes radio waves as the transmission medium. The power transfer in VLC systems such as SLIPT can be calculated using the VLC path loss model:

(4) $$P_{r}=\frac{\left(m+1\right)A_{d}{\left(h/d\right)}^{m}\cos^{m}\left(\phi \right)P_{t}}{2\pi d^{2}}.$$

where $P_{r}$ represents the received optical power, $A_{d}$ the detector physical area, *h* the height difference between transmitter and receiver, *d* the transmitter–receiver distance, ϕ the angle of irradiance, *m* the Lambertian order (a characteristic of the LED described by $m=-\ln2/\ln(\cos\left(\Phi_{1/2}\right))$, with $\Phi_{1/2}$ representing the transmitter semi-angle at half power), and $P_{t}$ the transmitted optical power.

Within the scope of CR networks, the fusion of SWIPT and SLIPT can notably elevate the performance of SUs by furnishing an auxiliary power source and ensuring efficient spectrum usage. However, such integration requires sophisticated algorithms and protocols to maintain a balance between information decoding and energy harvesting, and to avoid interference with PUs. The potential of SWIPT and SLIPT in CR networks is yet to be fully exploited, with research ongoing to surmount the challenges inherent in their implementation.

Supporting this, several papers [74,83,84] present various cognitive strategies pertinent to DSA, Spectrum Sensing, Spectrum Decision-Making, Spectrum Efficiency, and Spectrum Interference Mitigation. The objective is to optimize the performance of VLC and RF systems in terms of energy efficiency and information transfer rate. This involves proposing solutions to counterbalance the trade-off between information decoding and energy harvesting and to mitigate interference with PUs.

The research work [83] proposes a hybrid system integrating VLC and RF to augment data rate and reliability. This system features a cognitive-inspired resource allocation policy for a hybrid RF/VLC system, designed to fulfill the rate requirement of the VLC user while leaving surplus resources for the RF user.

Simultaneously, ref. [84] puts forth a dual-hop hybrid VLC and RF IoT system predicated on SLIPT. This system aims to support energy-constrained IoT devices in diverse indoor/outdoor environments. The study presents a dynamic balance between the information and energy flows over the system, with the optimal approach guaranteeing reliable information delivery by efficiently leveraging available energy.

Lastly, ref. [74] presents a cognitive-based RF/VLC system with SLIPT. It employs a cognitive-based resource allocation to enable information transmission from the VLC user to an RF user who cannot directly receive the information emitted from the VLC AP, without compromising the required QoS of the VLC user.

Despite these advancements, challenges remain. The system proposed in [83] demonstrates significant outages in both IoT and Licensed User (LU) networks, potentially disrupting communication. The suggestions in [74,84] do not explicitly address potential limitations, challenges, or drawbacks, which could be inferred from the complexity of the proposed systems, the assumptions made in the system models, or the constraints imposed by the cognitive-inspired resource allocation policies. Therefore, further work is necessary to assure the robustness of these systems and to explore solutions for their limitations.

Table 4 provides a comparative summary of the reviewed papers, highlighting the proposed systems, key features, problem formulation, and performance evaluation.

In conclusion, the integration of SWIPT and SLIPT into CR networks unveils a promising pathway for the evolution of wireless communication systems. These techniques potentially foster significant advancements in energy efficiency and spectrum utilization, marking a significant stride in the field of cognitive radio networks. However, it also introduces new challenges that necessitate further research. Future studies should focus on devising efficient algorithms and protocols that can adeptly balance the trade-offs between information decoding, energy harvesting, and interference avoidance with PUs. Moreover, the exploration of hybrid systems that integrate RF and VLC, along with the simultaneous transfer of information and power, remains a vital research area. Further research could involve conducting simulations and real-world tests of these hybrid systems, complemented by the development of performance evaluation metrics that can more accurately assess their effectiveness and efficiency. This endeavor is critical in meeting the growing demand for energy-efficient and high-capacity wireless networks.

### 5.7. Resource Allocation in Multiple Access

The efficient allocation of resources in hybrid communication networks, encompassing RF and VLC, is a subject of paramount importance. This subsection elucidates the application of cognitive strategies in the context of sensor networks, with a particular emphasis on Rate-Splitting Multiple Access (RSMA) and Non-Orthogonal Multiple Access (NOMA).

RSMA, a technique extensively studied in RF communications, has been adapted to optical and visible light systems. The constraints inherent to these signals, such as peak and average optical power limitations, must be meticulously considered in RSMA design and optimization. Analogous to RF systems, RSMA has been shown to surpass SDMA and NOMA in optical and visible light communications.

In the domain of VLC networks, RSMA has been portrayed as a versatile interference management scheme, demonstrating adaptability and superior performance in terms of Weighted Sum Rate (WSR) [89]. Research has also concentrated on amalgamating NOMA and Space-Division Multiple Access (SDMA) in MISO VLC channels, underscoring the potential of RSMA as a promising solution for indoor multi-cell VLC systems [78]. Further, the optimal beamformer design for RSMA-aided VLC networks has been explored, deriving the lower bound of achievable rates and maximizing the sum rate under optical and electrical power constraints [90].

A comparative summary of the reviewed papers on RSMA is presented in Table 5, highlighting key features, problem formulations, and results.

The inclusion of NOMA in this review is justified by its role as an effective technique to circumvent the shortcomings of VLC systems, as detailed in the paper “NOMA-Based VLC Systems: A Comprehensive Review” (NOMA2) [73]. NOMA’s potentials to increase the number of users, system’s capacity, massive connectivity, and enhance spectrum and energy efficiency in future communication scenarios have been highlighted. The integration of NOMA into VLC systems has been shown to minimize interference and enhance performance. Furthermore, the application of NOMA in MIMO-VLC systems, including certain power allocation strategies, has been demonstrated to improve the achievable sum rate [73].

In conclusion, the cognitive strategies encompassed by RSMA and NOMA in the context of sensor networks utilizing hybrid RF/VLC systems present a multifaceted approach to resource allocation, while RSMA may outperform NOMA in specific scenarios, the study of both techniques offers a comprehensive understanding of multiple access techniques. The choice between NOMA and RSMA may ultimately hinge on the specific requirements, constraints, and objectives of a particular system or application, thereby necessitating a nuanced and cognizant approach to their implementation.

### 5.8. Machine Learning

The integration of ML techniques into VLC and hybrid RF/VLC systems has emerged as a significant research domain. Paper [86] provides an extensive survey on ML algorithms in CRSNs, with a focus on spectrum sensing, dynamic spectrum access, and associated challenges and solutions. Paper [85] introduces a reinforcement learning approach for hybrid WiFi-VLC networks, utilizing a Q-learning algorithm to augment network capacity, and showcasing enhancements in throughput and fairness. Paper [81] investigates the amalgamation of VLC and Device-to-Device (D2D) technologies into heterogeneous networks through Q-learning, optimizing data transmission routes. Paper [87] details a CNN-based demodulator for NOMA-VLC, emphasizing spectral efficiency and distortion mitigation. Paper [88] may further elucidate ML applications in VLC and hybrid RF/VLC systems. Collectively, these studies highlight the potential of ML in augmenting the performance, efficiency, and resilience of VLC systems, indicating promising avenues for future exploration.

The application of ML in cognitive strategies extends to various aspects of sensor networks, as detailed below:

Spectrum sensing: ML aids in detecting white spaces and controlling interference, employing techniques such as Q-learning.

Dynamic spectrum access: game theory and reinforcement learning are utilized for efficient spectrum management.

Resource management: ML assists in managing resources to meet QoS requirements through algorithms such as genetic algorithms and reinforcement learning.

Channel estimation and spectrum prediction: algorithms such as hidden Markov models and LSTM are frequently used.

Cooperative spectrum sensing: ML enhances cooperative spectrum sensing in CR networks through Bayesian machine learning and other techniques.

Interference control: ML assists in controlling interference using distributed Q-learning.

Channel identification: techniques such as Q-learning and KNN aid in channel identification.

Spectrum management: ML aids in spectrum management through Q-learning and artificial bee colony optimization.

Radio scene analysis: techniques such as reinforcement learning are used for radio scene analysis.

Spectrum allocation and spectrum auction: ML assists in spectrum allocation and auction through game theory and other methods.

Power control and resource allocation: techniques such as Q-learning and artificial neural networks are employed for power control and resource allocation in CR networks.

The papers reviewed cite some drawbacks and challenges in the integration of ML techniques in VLC and hybrid RF/VLC systems. Examples of these challenges include the complexity of implementing real-time models, the need for algorithms to converge within limited time frames, potential scalability issues in hybrid networks, the absence of extensive real-world testing to validate certain approaches, and potential computational costs in applying advanced techniques such as CNN-based demodulators. Collectively, these studies emphasize the necessity to carefully consider these practical challenges and complexities, underlining areas that require further exploration and refinement in the application of ML to VLC and hybrid RF/VLC systems.

Table 6 provides a comparative summary of the reviewed papers, encapsulating the proposed systems, key features, problem formulations, and performance evaluations, further illustrating the integration of ML techniques in VLC and hybrid RF/VLC systems.

## 6. Discussion

The integration of cognitive hybrid RF/VLC systems with emerging technologies such as 5G, 6G, and IoT is a pivotal area of research, promising to enhance user mobility and optimize network capacity [64]. The exploration of innovative network topologies and performance analyses is vital, particularly in the context of the 6G era, to further optimize the hybrid RF/VLC systems [31].

The synergy between cognitive hybrid RF/VLC systems and IoT communication strategies stands as a significant development. Recent studies underscore the necessity of robust communication strategies in fostering an efficient smart environment. A systematic review in [101] delineates various IoT communication strategies that have been instrumental in enhancing the interconnectivity between applications, users, and smart devices, categorized into device-to-device, device-to-cloud, device-to-gateway, and device-to-application scenarios. This structured approach facilitates seamless communication in IoT environments, offering a pathway to effectively integrate these strategies into cognitive hybrid RF/VLC systems.

Furthermore, energy-aware strategies in hybrid RF/VLC systems, especially in device-to-device (D2D) communication, have emerged as a crucial research direction. Paper [102] highlighted the significance of energy-aware hybrid RF-VLC multiband selection in D2D communication, emphasizing the role of such strategies in optimizing energy consumption and enhancing communication efficiency.

The application of machine learning and deep learning techniques is also emerging as a significant area of research, potentially addressing the challenges associated with high data rates and ensuring QoS [86]. The potential of Optical Camera Communication (OCC) in beyond-5G applications, especially in the context of IoT, represents a promising research direction, focusing on vehicular communication and indoor applications [65].

Security and privacy remain paramount concerns in the development of cognitive hybrid RF/VLC systems. Developing strategies that leverage the high security features of optical wireless communication technologies is essential [66]. Moreover, interference management is a critical area, necessitating the development of effective strategies, possibly leveraging machine learning techniques, to manage the dynamic nature of wireless environments [63].

Standardization of protocols and frameworks is essential to facilitate interoperability and scalability in hybrid networks, covering aspects such as network architectures and modulation schemes [62]. Additionally, addressing the increased complexity associated with the integration of multiple technologies, including the development of seamless handover mechanisms and optimum resource allocation, remains a significant focus area [64]. Lastly, ensuring QoS, particularly in the high data rate requirements of 5G and beyond technologies, emerges as a vital area of research [31].

## 7. Conclusions

This work offers a comprehensive review of the advancements and potential avenues in the domain of CRSNs, particularly focusing on hybrid RF/VLC systems. The study highlights significant areas such as Energy Efficient Resource Allocation, Industrial Scenarios, and the application of Machine Learning techniques, identifying promising avenues to enhance the communication capabilities of hybrid systems.

While the study acknowledges the existing challenges and complexities, it emphasizes the necessity for further research and exploration in this field. The integration of emerging technologies, including ambient light harvesting and artificial intelligence, stands as a promising approach to fostering innovative solutions in wireless communication systems.

## Figures and Tables

**Table 1:** Comparative summary of review papers on hybrid RF/VLC systems.

Paper	Discussion and Focus	Strengths	Limitations
[62]	Comprehensive survey on hybrid wireless networks, covering both RF and VLC technologies.	Extensive coverage of hybrid wireless networks. Detailed discussion on network topologies and performance metrics.	Does not delve into the specific challenges and opportunities in the context of wireless sensor networks.
[63]	Focuses on hybrid LiFi and Wi-Fi networks. Discusses design considerations, performance metrics, and applications like IoT.	Detailed framework of system design. Discusses key performance metrics and applications.	Lacks a discussion on the adaptability and reconfigurability of the systems.
[31]	Detailed survey of hybrid RF/VLC systems, discussing network topologies, performance analyses, and applications.	Comprehensive overview of hybrid RF/VLC systems. Discusses both benefits and limitations of the technology.	Does not address the energy efficiency challenges that are critical for wireless sensor networks.
[64]	Discusses hybrid VLC and RF systems, focusing on access frameworks and application scenarios.	Detailed discussion on access frameworks. Addresses challenges in resource allocation and handover.	Does not discuss the scalability issues that could arise when deploying these systems in more complex environments.
[65]	Proposes a hybrid RF/VLC network architecture for IoT, particularly in remote areas.	Addresses the challenge of providing IoT connectivity in remote areas. Explores the interoperability of different communication technologies.	Does not discuss the robustness and resilience of the proposed architecture.
[66]	Comprehensive state-of-the-art study of hybrid VLC/RF networks. Discusses indoor and outdoor scenarios, and applications in IoT.	Extensive coverage of both indoor and outdoor scenarios. Discusses applications in IoT and future challenges.	Lacks a focus on the trade-offs between performance and complexity in hybrid systems.

**Table 2:** Potential areas for RF/VLC network improvement by CR strategies.

Topic	References
Energy-efficient resource allocation	[65,69,70,71,72,73,74,75,76,77,78,79]
Communication in industrial scenarios	[65,67,72,80,81]
Using M-MIMO	[73,82]
SWIPT (simultaneous wireless information and power transfer)	[74,83,84]
Artificial intelligence (AI)-based	[61,69,72,73,81,85,86,87,88]
Energy harvesting and management	[65,69,72,74,83,84]
Resource allocation in multiple access	[69,73,78,86,87,89,90]

**Table 3:** Comparative summary of resource allocation.

Paper	System Proposed	Performance Analysis	Metrics	Type of Problem Formulation
[76]	Hybrid RF/VLC system for energy-efficient wireless access.	Competitive ratio of O(log(M)log(N)).	Total power consumption of the system.	Optimization problem using ILP.
[75]	Hybrid RF/VLC with shared backhaul and imperfect CSI.	Two-layer approach using KKT conditions and Lagrange multipliers.	Weighted proportional fairness.	Convex optimization for joint resource allocation.
[70]	Hybrid RF/VLC network for energy-efficient resource allocation.	Evaluated via simulations.	Energy efficiency, total data rate, and sum rate.	Concave-convex fractional program, using KKT conditions.
[72]	Heterogeneous RF/VLC system for URLLC in Industrial IoT networks.	Evaluated via simulations using PDS-ERT.	Energy efficiency, reliability, latency, and data transmission rates.	Decision-making problem, modeled as an MDP.
[71]	Multi-cell cognitive VLC model with a hybrid underlay/overlay power assigning strategy.	Simulations, focusing on sum-rate and area spectral efficiency.	Sum-rate, area spectral efficiency, and Field of View (FoV) angle.	Optimization problem to maximize spectral efficiency of VLC systems.
[65]	Hybrid RF/VLC network architecture for IoT, leveraging solar-powered lighting systems.	Validated through fragmentation, in-depth VLC tests, and real-scenario experiments.	Success rate of fragmentation, Bit Error Rate (BER), error probabilities, propagation times, and transfer times.	Need for high connectivity and interoperability in IoT systems.
[79]	5G network with passive reflectors for enhanced connectivity.	Evaluated via simulations demonstrating improved connectivity and energy efficiency.	SNR and total power consumption.	Optimization problem focusing on maximizing SNR and minimizing power consumption.

**Table 4:** Comparative Summary on SWIPT and SLIPT.

Paper	Proposed System	Key Features	Problem Formulation	Performance Evaluation
[83]	Hybrid RF/VLC system with SLIPT and SWIPT protocols.	Two-way cooperative communications.	Outage minimization for LU and IoT networks.	Based on outage probabilities and throughput.
[84]	Dual-hop hybrid RF/VLC IoT system with SLIPT.	Evaluated via mathematical analyses and simulations	PDF of Harvested Energy at the relay node, and the relationship between its lower and upper thresholds	Dynamic balance between information and energy flow.
[74]	Hybrid RF/VLC system with SLIPT.	Cognitive-based resource allocation to serve RF user without burdening VLC user.	Optimization problem to maximize harvested energy under constraints.	Performance evaluated in terms of outage probability and harvested energy.

**Table 5:** Comparative summary of RSMA.

Paper	Proposed System	Key Features	Problem Formulation	Results
[89]	RSMA in VLC to improve sum rate.	Joint power allocation and beamforming design.	Non-convex optimization problem, transformed using SCA.	Outperforms traditional NOMA and OMA in sum rate.
[78]	RSMA in VLC for energy efficiency.	Joint user pairing and power allocation.	Non-convex optimization problem, solved using bisection search.	Achieves better energy efficiency compared to traditional NOMA and OMA.
[90]	RSMA in VLC with optimal beamformer design.	Derives the lower bounds of the achievable rate of each user	Sum rate maximization under power constraints.	Superior performance compared to several baseline schemes.

**Table 6:** Comparative summary of ML applied to hybrid RF/VLC systems.

Paper	Proposed System	Key Features	Problem Formulation	Performance Evaluation
[86]	ML Algorithms for Applications in CRSNs	Discusses ML for spectrum sensing, auction, prediction in DSA applications.	Focuses on CRN challenges, robustness, scalability.	Overview of advancements in ML for CR.
[85]	Reinforcement Learning (RL) for Hybrid WiFi-VLC Networks	Centralized Q-learning algorithm, new reward function considering user location.	Maximize system throughput by reassigning users to APs.	Numerical results show improvement in throughput, fairness.
[81]	VLC and D2D Heterogeneous Network Optimization using RL	Single-agent Q-learning for optimal routing, maximizing rewards.	Single-agent RL scenario for optimal routing in VLC-D2D network.	Details Q-learning algorithm, no specific performance results.
[87]	CNN-Based Signal Demodulator in NOMA-VLC	CNN-based demodulator to mitigate linear and nonlinear distortions.	Focuses on error propagation, multipath distortions, nonlinearity in NOMA-VLC.	Simulation and experiment results show mitigation of distortions, improved performance.

## Abbreviations

The following abbreviations are used in this manuscript:

AP	Access Point
BER	Bit Error Rate
CR	Cognitive Radio
CRLB	Cramer–Rao Lower Bound
CRSNs	Cognitive Radio Sensor Networks
CSI	Channel State Information
DCO-OFDM	Direct-Current-Biased Optical Orthogonal Frequency Division Multiplexing
DSS	Dynamic Spectrum Sharing
DSA	Dynamic Spectrum Access
D2D	Device-to-Device
FoV	Field of View
HetNets	Heterogeneous Networks
KKT	Karush–Kuhn–Tucker
kNN	k-Nearest Neighbors
LU	Licensed User
ILP	Integer Linear Programming
IoT	Internet of Things
LoS	Line-of-Sight
MDP	Markov Decision Process
MDPI	Multidisciplinary Digital Publishing Institute
m-MIMO	Massive Multiple-Input, Multiple-Output
MIMO	Multiple-Input, Multiple-Output
NLoS	Non-Line-of-Sight
NOMA	Non-Orthogonal Multiple Access
OCC	Optical Camera Communication
PCA-kNN	Principal Component Analysis with k-Nearest Neighbors
PCA-NN	Principal Component Analysis with Neural Network
PSO	Particle Swarm Optimization
PUs	Primary Users
QoS	Quality of Service
RSMA	Rate-Splitting Multiple Access
RF	Radio Frequency
RMS DS	Root Mean Square Delay Spread
SLIPT	Simultaneous Lightwave Information and Power Transfer
SNR	Signal-to-Noise Ratio
SPIE	The International Society of Optics and Photonics
SUs	Secondary Users
SWIPT	Simultaneous Wireless Information and Power Transfer
URLLC	Ultra-Reliable Low-Latency Communications
VLC	Visible Light Communication
WSNs	Wireless Sensor Networks
